# A cross-sectional study on peroneal muscle echogenicity changes and their effects on balance functions in individuals with chronic ankle instability

**DOI:** 10.1038/s41598-025-00175-3

**Published:** 2025-04-29

**Authors:** Cheryl Shu Ming Chia, Sai-Chuen Fu, Violet Man-Chi Ko, Ming Wang, Yuxin Zuo, Patrick Shu-Hang Yung, Samuel Ka-Kin Ling

**Affiliations:** 1 Department of Orthopaedics and Traumatology, Faculty of Medicine, The Chinese University of Hong Kong (CUHK), Hong Kong SAR, China; 2 Office of Graduate Studies and Professional Learning, National Institute of Education, Nanyang Technological University, Singapore, Singapore

**Keywords:** Ankle instability, Peroneal muscle, Postural control stability, Dynamic balance, Musculoskeletal system, Anatomy, Diseases, Medical research, Pathogenesis, Health care, Diagnosis, Disease prevention, Medical imaging, Prognosis

## Abstract

This study investigated the relationship between peroneal muscle echogenicity and balance function in individuals with chronic ankle instability (CAI). While prior research has examined peroneal muscle activity, reaction time, and balance, the impact of echogenicity—an indicator of myosteatosis/fibrosis—remained underexplored. Cross-sectional study. Sixty-two adults with CAI were included. Peroneal muscle size, echogenicity, and stiffness were assessed using ultrasound. Dynamic balance was evaluated via the Y balance test (YBT), and static postural control was evaluated during lateral step-down (LSDT) and single-leg stance test (SLST). Eversion strength was assessed with a dynamometer. The relationship between muscle characteristics and balance was assessed using canonical correlation and stepwise linear regression. Individuals with increased peroneal muscle echogenicity had reduced muscle size, poorer eversion strength, and poorer balance. Eversion strength is positively associated with YBT scores across all echogenicity levels and negatively associated with posture parameters during the LSDT in moderate echogenicity. Peroneal longus stiffness was positively associated with YBT in severe echogenicity and posture parameters during the SLST. Increased peroneal muscle echogenicity is associated with poorer eversion strength and stiffness, resulting in poorer balance performance. Improving the peroneal muscle quality may enhance functions in the CAI condition.

**Supplementary Information:**

The online version contains supplementary material available at 10.1038/s41598-025-00175-3.

## Introduction

Lateral ankle sprains (LAS) are a common soft tissue injury that can lead to Chronic Ankle Instability (CAI)^1,2^. Up to 40% of individuals with an acute LAS progress to CAI due to injury severity, structural deformity, and inadequate rehabilitation^3,4^. The prevalence of CAI constitutes 25%, ranging between 7% and 53% in different populations^5,6^. CAI is characterised by recurrent ankle sprains, perception of the ankle giving way, persistent pain, muscle weakness, and impaired postural control, restricting exercise and impacting the quality of life^7–9^. Therefore, an adequate and early treatment is essential to avoid the condition’s progression.

The function of the peroneal muscles, which play a crucial role in maintaining ankle joint stability, is among the key factors in influencing CAI progression. Peroneal muscles contribute to eversion strength and are pivotal in maintaining ankle joint stability, especially in the sagittal and frontal planes after a LAS event. They are the main peri-ankle structure that compensates for the impaired functions of the various lateral ligaments^10^. However, the changes in the peroneal muscle quality (including the presence of myosteatosis and fibrosis) remain understudied and may influence eversion strength^10,11^. According to previous literature, post-LAS events may change the peroneal muscle architecture where increased adipose tissue correlates with an increased frequency of ankle sprains and exacerbate balance issues^12,13^. However, only two studies have investigated the presence of myosteatosis/ fibrosis in the muscles in a small sample size of individuals experiencing an acute LAS. In other types of musculoskeletal injuries, such as ACL injuries and rotator cuff tears, it has been observed that a decrease in muscle size, along with the formation of fibrotic tissue and fatty tissue deposition, limits force generation as less area of the muscle is occupied by contractile components^14–18^. The increase in fibrotic tissue and adipose tissue deposition in the skeletal muscle could alter the pennation angle and fascial length and decrease force production^19^. Consequently, this affects the physical functions^18,20^. In addition, an inverse relationship between the central activation ratio during an isometric contraction and intramuscular fat/fibrosis tissue accumulation in skeletal muscle was identified in other population groups that have high muscle echogenicity (e.g. elderly population)^21^. It has been highlighted that the increase in IMAT in the quadricep muscle is associated with delayed or incomplete motor unit recruitment, hence the muscles may not activate efficiently^22^. The decreased ability of muscle activation may impair an individual’s ability to recover from a balance perturbation^21^. Hence, altering the amount of adipose tissue/fibrosis in the muscle is associated with both improving neuromuscular activation and altering the proprioception through mechanical changes^23^. This could reduce posture control sway and balance function^23^.

In addition, an increased passive stiffness in CAI is observed to stabilise the ankle and foot, possibly due to an increase in peroneal longus activity after an ankle inversion, resulting in an eccentric strain injury^24–28^. Muscle stiffness reflects the ability of the structures around the joints to resist passive displacement of the joint, with either inadequate passive stiffness or excessive passive stiffness leading to poor clinical outcomes^29,30^. Therefore, investigating the changes in passive stiffness during post-injury can aid clinicians in monitoring the capsuloligamentous tissue alterations effectively. Currently, limited evidence exists regarding the relationship between muscle quality and stability among the CAI population; hence, evaluating whether poorer peroneal muscle quality affects balance functions may provide insights for future targeted treatment of the muscle^31–33^.

The current conservative treatment effect remains heterogeneous as the training may not specifically improve muscle quality as the effectiveness of the conservative treatment varies and does not address the full continuum of impairment and disability^34–40^. For instance, the improvement of muscle quality (e.g. muscle echogenicity) can be hindered by the significant loading from the strengthening rehabilitation programme^41^. The resistance band training performed with a band tied in a neutral position may not mechanically strengthen the peroneal longus muscle as the muscle is associated with eversion in ankle plantarflexion^42^. This is further supported by a systematic review conducted by Luan et al.^36^ which found that the rigidity and unvaried strengthening rehabilitation is not effective in mitigating symptoms associated with the CAI condition. Concurrently, systematic reviews have highlighted that balance training may not be effective in restoring static balance^38,39^. Therefore, evaluating the quality status of the peroneal muscles may be essential to prevent the development of CAI.

This cross-sectional study aimed to investigate if increasing the peroneal muscle echogenicity has smaller peroneal muscle size, higher passive stiffness, poorer strength, and balance function. In addition, to explore the relationship between muscle strength and stiffness with various balance assessments according to the echogenicity gradings. We hypothesised that higher peroneal muscle echogenicity is associated with weaker muscle strength, smaller peroneal muscle cross-sectional area, higher passive stiffness and poorer balance functions.

## Methods

### Study design

This cross-sectional study was conducted in compliance with the Declaration of Helsinki and was approved by The Joint Chinese University of Hong Kong- New Territories East Cluster Clinical Reseach Ethics Committee (Ethics approval number: 2022.263). The Strengthening the Reporting of Observational Studies in Epidemiology (STROBE) initiative was adopted. Patients with CAI were recruited from a local hospital between June 2023 and January 2024.

### Inclusion and exclusion criteria

Clinical diagnoses were made by the Foot and Ankle surgeons per the International Ankle Consortium^24^. The recruited patients were aged between 18 and 50 years old with a history of at least one significant inversion ankle sprain, in which the initial sprain must have occurred at least 12 months before study enrolment. The sprain had to be associated with inflammatory symptoms and created at least one interrupted day of desired physical activity, with the recent sprain occurring more than 3 months before study enrolment. Participants should report at least two episodes of giving way in the 6 months before study enrolment. Furthermore, patients were screened with the self-reported ankle instability, Cumberland Ankle Instability Tool (CAIT) < 20.5 (Cantonese)^43^, which had been validated in the Hong Kong population with a high sensitivity of 0.900 and high specificity of 0.860. Lastly, the surgeon had to evaluate the patients with a positive anterior drawer test. Patients were excluded if they had reported a history of fractures, dislocations, or surgeries of the lower extremities, any lower extremity neuromusculoskeletal abnormalities, or neurological disorders. X-rays and ultrasounds were arranged by the surgeon for the patients to ascertain the absence of fractures or any abnormalities in their lower extremities. Written informed consent was obtained from all participants. G ^*^ Power3.1.9.2 statistical software (NeuIsenburg, Aichach, Germany)^44^ calculated the sample as 62, based on the multiple linear regression model using the calculated effect sizes from our study between 0.21 and 0.54, β of 0.2 and α of 0.05.

### Measures

CAIT was used to measure perceived instability, consisting of nine questions including a question on ankle pain, and questions on feeling ankle instability. The score ranged from 0 to 30 with a greater score indicating higher stability^43,45^.

The YBT (FMS Y balance) evaluated the dynamic balance^46–49^. Participants began with their non-injured limbs first before progressing to their injured limbs^50^. The relative length (cm) of the lower limb was measured (anterior superior iliac crest-medial malleolus). The formula for the normalized maximal reach distance is the longest distance in each direction divided by the participant’s leg length multiplied by 100^51^.

Tekscan Matscan ^®^ pressure mat model 3150 was used to evaluate postural control^52,53^. The lateral step-down test (LSDT) stimulates stair-like movement where the participants step down from the 30 cm high block and balance unilaterally for 20 s on the pressure mat^54^. However, the data obtained from the Tekscan pressure mat was initiated after the beginning of the study, resulting in an incomplete baseline data set for this variable.

For the single-leg stance test (SLST), participants were instructed to balance on the pressure mat for 20 s with their eyes opened and closed^55,56^. The sway values were analysed by the Sway Analysis Module (SAM™).

An isometric digital handheld dynamometer (MicroFET2, Manual Muscle Testing) was used to measure eversion strength^57^. A handheld dynamometer was placed on the lateral 5th metatarsal head and the patient exerted the maximum eversion power for 5 s isometrically. The maximum strength was taken as the participant’s peak strength and was normalised by the body weight (kg). The inter-rater operator measurement had an intraclass correlation coefficient (ICC) of 0.942, and a 95% confidence interval (CI): of 0.806–0.983. The intra-operator measurement between sessions was ICC: 0.917, 95% CI: 0.621–0.981).

An ultrasound system (Supersonic Imagine’s Aixplorer, Aix-en-Provence, France) with a linear transducer array (5-18 MHz; SuperLinear 18 − 5) was used to measure the cross-sectional area (CSA) of the peroneal muscles, stiffness and echogenicity, Appendix 1. According to the SENIAM recommendations, the probe was placed at the upper ¼ position between the tip of the head of the fibula and the tip of the lateral malleolus of the patient^58,59^. Markings were made on the measurement points for reproducibility. After applying sufficient gel to the probe, the probe was placed perpendicular to the markings with minimal force^60^. To minimise the effect of the ultrasound anisotropy, the probe was held in a maximally vertical position to capture the peroneal muscles in a short-axis view^60^. At the same position, a region of interest with an analysis area of a 5 mm diameter circle was set near the centre at the peroneal longus to measure the passive stiffness, Appendix 2^61^. The transducer was placed for < 10 s to confirm the shear elastic modulus at a stable colour distribution. Since the skeletal muscle is not assumed to be isotropic, the shear modulus is reported as a young modulus divided by 3^62^. For the ultrasound measurements, the inter-operator measurement was conducted for the CSA 25% (ICC: 0.929, 95% CI: 0.669–0.986) and young modulus (ICC: 0.935, 95% CI: 0.677–0.987). The intra-operator between sessions for CSA 25% was reported (ICC: 0.860, 95% CI: 0.383–0.979), and young modulus (ICC: 0.912, 95% CI: 0.609–0.980). Visual assessment using the Modified Heckmatt Scale (MHS)^63,64^ was used to categorise muscle echogenicity by the trained researcher and a physiotherapist independently to minimize any subjectivity. The four different types of grading^65^ can be seen in Table 1. The inter-rater was 0.93 (95% CI: 0.89–0.95) and the intra-class correlation was 0.92 (95% CI: 0.89–0.95).

**Table 1:** Different grading of the modified heckmatt scale.

Grading	Modified Heckmatt scale
1	Normal echogenicity with more than 90% of the muscles that is distinct from bone echo
2	Increased muscle echogenicity in 10–50% of the tissue, distinct bone and area of the normal muscle echo
3	Increased muscle echogenicity between 50–90% of tissue with reduced distinction of bone echo from muscle
4	Very strong muscle echogenicity with near complete loss of distinct bone echo from muscles > 90% of tissue.

### Statistical analysis

The characteristics of the study population were presented as the median(inter-quartile range) or frequency (n)%. The distribution of the quantitative data was assessed by the Kolmogorov-Smirnov test (*p* > 0.05). Peroneal muscle quality and balance functions were compared by the echogenicity grading using one-way ANOVA, and presented as median (IQR). Posthoc analysis with Bonferroni correction was conducted for the different echogenicity grading. The distribution of the quantitative data was assessed by Kolmogorov-Smirnov test (*p* > 0.05). Canonical correlation analysis was employed to examine the relationship between peroneal muscle quality and balance functions. Canonical loading was generated to link between the original and canonical variables. Variables with a loading > 0.3 were deemed significant for the variables of interest^66^. Hence, the total composite score of YBT, area of the centre of pressure (COP) during SLST (eyes opened), and anteroposterior/mediolateral excursion sway during LSDT were selected as a representative for each outcome to be included in the stepwise linear regression, stratified according to the grades of echogenicity. This was to identify the influence of muscle echogenicity on strength and stiffness affecting balance functions with the inclusion of confounding factors such as age, gender, and body mass index (BMI). Multiple imputation by chained equations with five different iterations was conducted to address the missing data for the postural control parameters during LSDT among individuals who were classified in the MHS group 4. No sensitive analysis, but sub-group analysis was conducted instead. Variance inflation factors exceeding 10 were checked to address multicollinearity. Please refer to Appendix Table 2 for the relevant VIF values reported. All statistical tests were conducted using a two-sided 5% significance level using SPSS 29.0 (IBM).

## Results

**Fig. 1:**
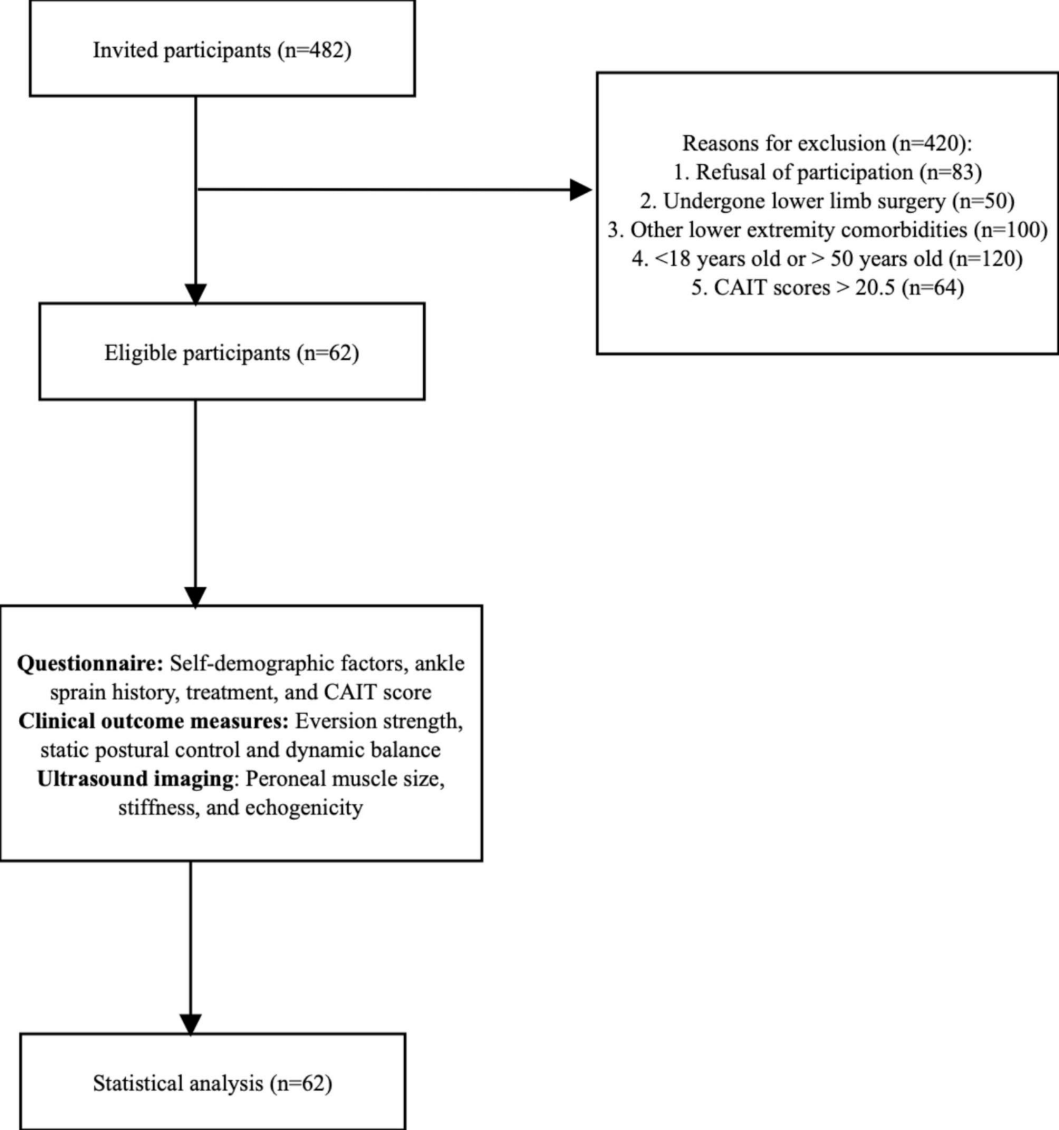
Enrolment diagram.

A total number of *n* = 482 were screened for eligibility between June 2023 to January 2024 in a local hospital by trained research assistants. However, due to the exclusion criteria such as refusal to participate after explaining the procedures (*n* = 83), individuals who had previous lower limb surgeries (*n* = 56), acute lower limb injuries (*n* = 97), being < 18 years old or > 50 years old (*n* = 120), and having CAIT scores > 20.5 (*n* = 64) were excluded. Finally, a total of *n* = 62 participants (40 females, and 22 males) who answered questionnaires on self-demographic factors, medical history, and CAIT scores, and performed clinical outcome measures together with ultrasound imaging were included for analysis (Fig. 1).

The median age of the population is 29 (23, 37) years old, with a median BMI of 22.2 (20.5, 25.4) kg/m^2^. The median duration of the last ankle sprain experienced was 36.0 (16.5, 60.0) months, with mild pain at the ankle during exercise experienced with a median visual analog scale of 25.0 (10.45, 75). The median score for CAIT for the affected limb among patients with unilateral ankle sprain was 13.5 (11.0, 18.0) meanwhile the self-reported CAIT score for the affected limb among patients with bilateral ankle sprain was 18.0 (13.5, 20.5). More than 50% of patients with CAI experienced ankle instability despite prior rehabilitation treatment (Table 2).

**Table 2:** The demographic characteristics of 62 patients with CAI.

Variables	Data
Age (years)	29 (23, 37)
BMI (kg/m^2^)	22.2 (20.5, 25.4)
Gender F (%)	40 (64.52)%
Height (m)	1.65 (1.61, 1.70)
Weight (kg)	60.50 (52.75, 73.00)
CAIT score	
Unilateral (affected side)	13.5 (11.0, 18.0)
Unilateral (least/not affected side)	29.5 (26.3, 30.0)
Bilateral (affected side)	12.0 (6.5, 17.0)
Bilateral (least/not affected side)	18.0 (13.5, 20.5)
CAI condition	
Unilateral (%)	28 (45.2%)
Bilateral (%)	34 (54.8%)
Duration of last ankle sprain history (months) (affected side)	36.0 (16.5, 60.0)
Any previous conservative treatment Y(%) (affected side)	38 (61.3%)
VAS-pain at the ankle during exercise (affected side)	25 (10.45, 75)

Data presented in median (Median (IQR) or n(%)), unless otherwise stated.

**Fig. 2:**
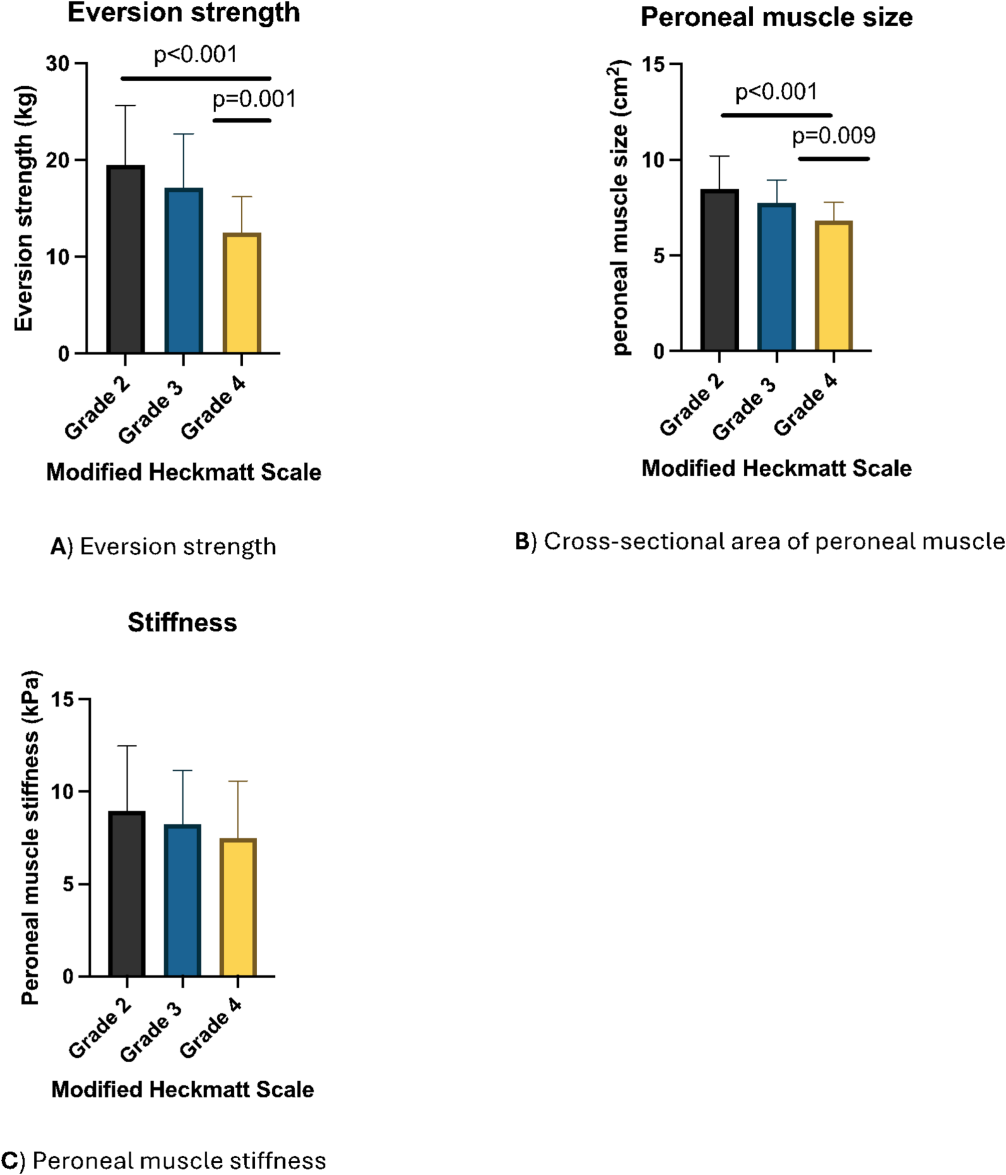
Peroneal muscle characteristics based on Modified Heckmatt Scale classification. (**A**) Eversion strength, (**B**) Cross-sectional area of peroneal muscle, (**C**) Peroneal muscle stiffness.

Eversion strength demonstrated a significant difference across various echogenicity grading (*p* < 0.001) with grade 2 of the echogenicity exhibiting the highest strength (19.89 kg, SD = 6.01), while grade 4 had the lowest (12.48 kg, SD = 3.73) (Fig. 2). A significant difference was observed in the CSA of the peroneal across the echogenicity grading (*p* < 0.001). Grade 2 had the biggest CSA (8.47 cm², SD = 1.74), while grade 4 had the smallest CSA of the peroneal muscle (6.80 cm², SD = 0.97). No significant difference was identified in peroneal longus stiffness across the echogenicity grading (*p* = 0.244).

**Fig. 3:**
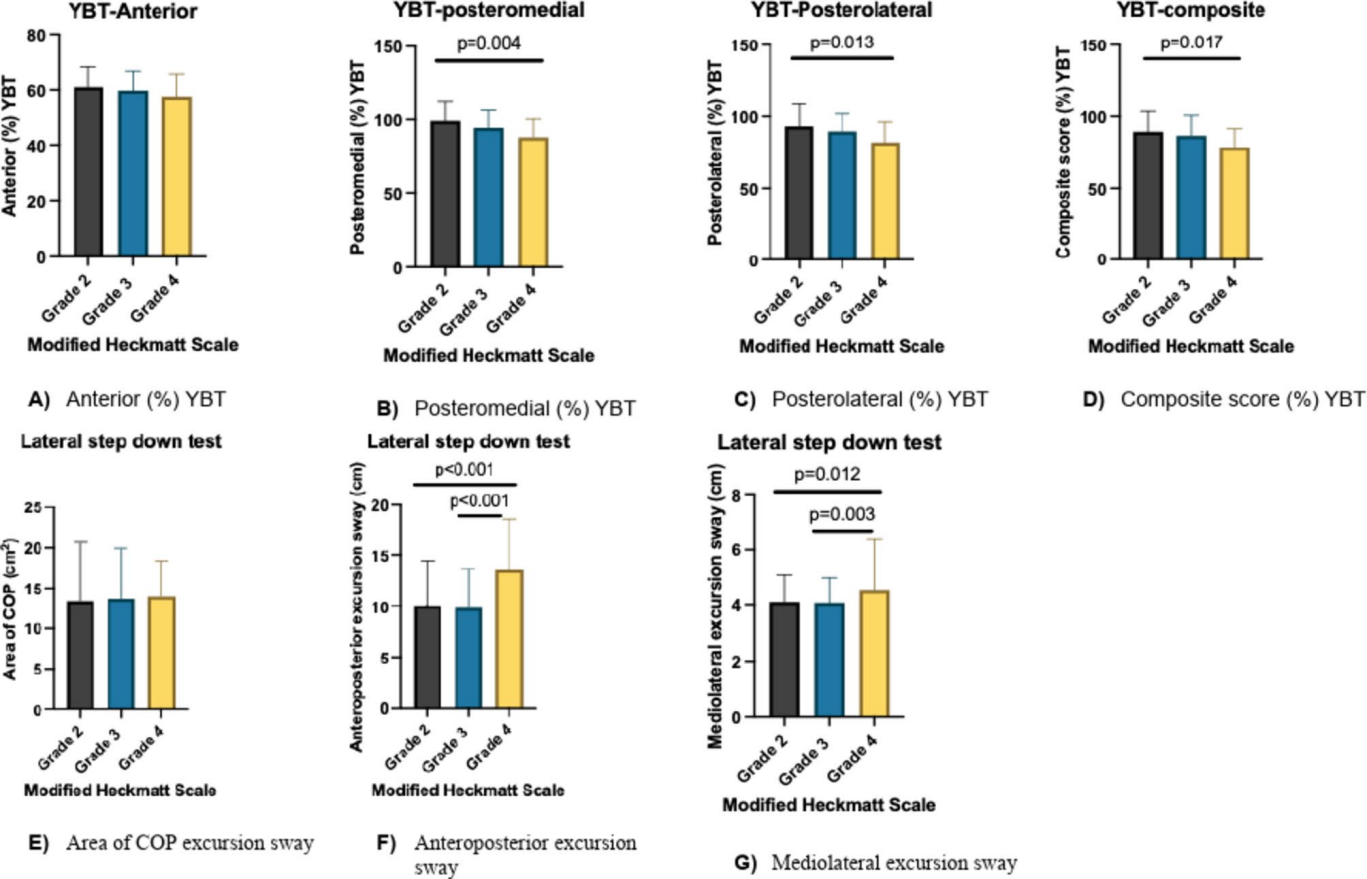
Dynamic balance and lateral step-down test based on modified Heckmatt scale classification.

**Fig. 4:**
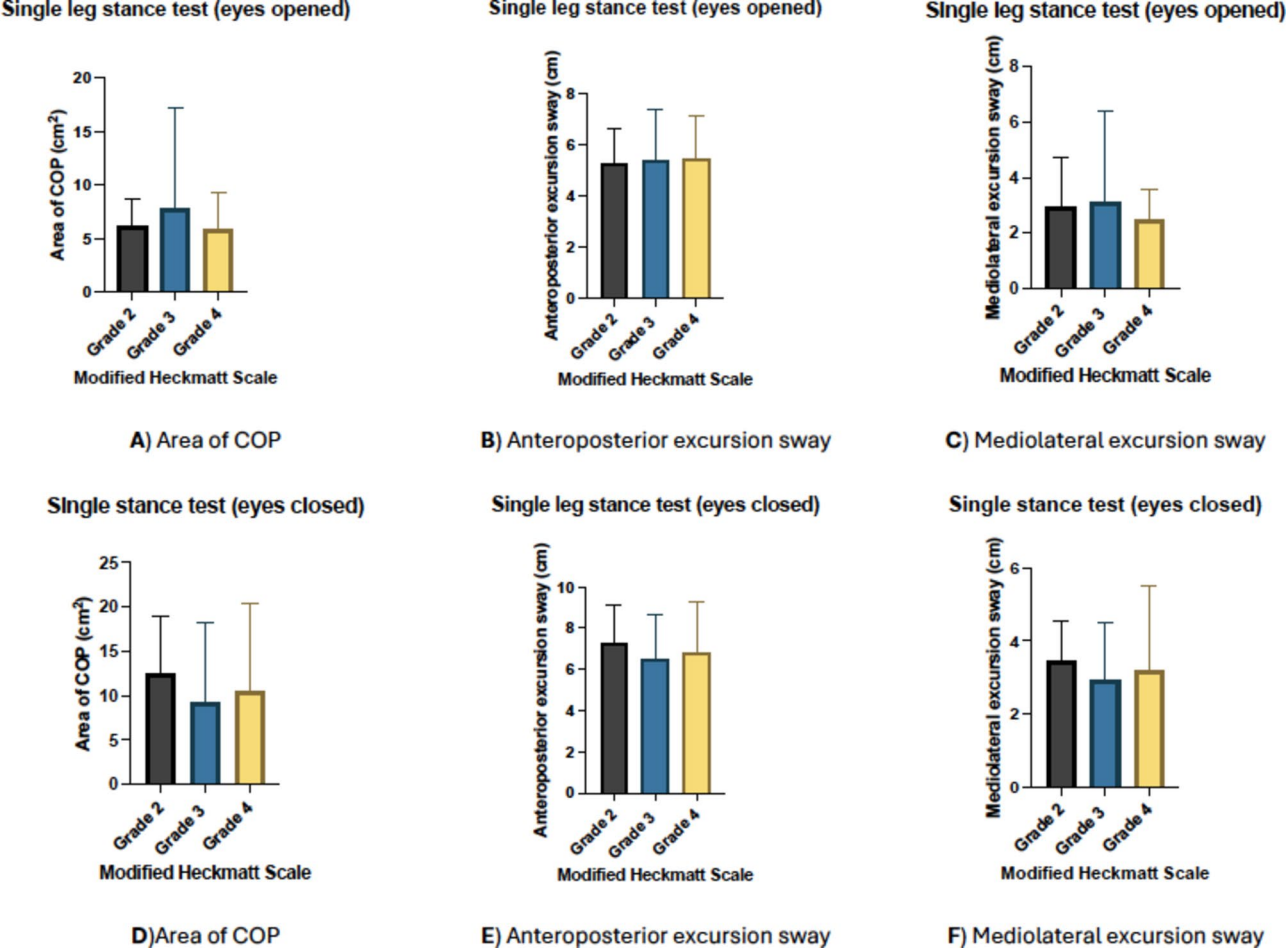
Postural control parameters according to modified Heckmatt classification.

For the LSDT (Fig. 3), a significant difference was observed in the anterior-posterior excursion sway between grades 2 and 4, with a mean difference of -3.52 (95% CI: -4.404, -2.640), *p* < 0.001). Moreover, a significant difference was observed between grades 3 and 4, with a mean difference of -3.82 (95% CI: -4.77, -2.88), *p* < 0.001). In addition, a significant difference was found in the mediolateral excursion sway between grades 2 and 4, with a mean difference of -0.40 (95% CI: -0.725, -0.066), *p* = 0.012). Additionally, a significant difference was observed between grades 3 and 4, with a mean difference of -0.41 (95% CI: -0.706, -0.115), *p* = 0.003). No significant differences were observed in the area of COP excursion sway during the LSDT. Across the Y balance test measures, a significant difference was found in the posteromedial (%) (*p* = 0.004), posterolateral (*p* = 0.013), and composite scores (*p* = 0.014) between grade 2 and grade 4. No significant differences were found in anterior (%) scores (*p* = 0.570) and between the sway parameters during SLST (Fig. 4).

**Fig. 5:**
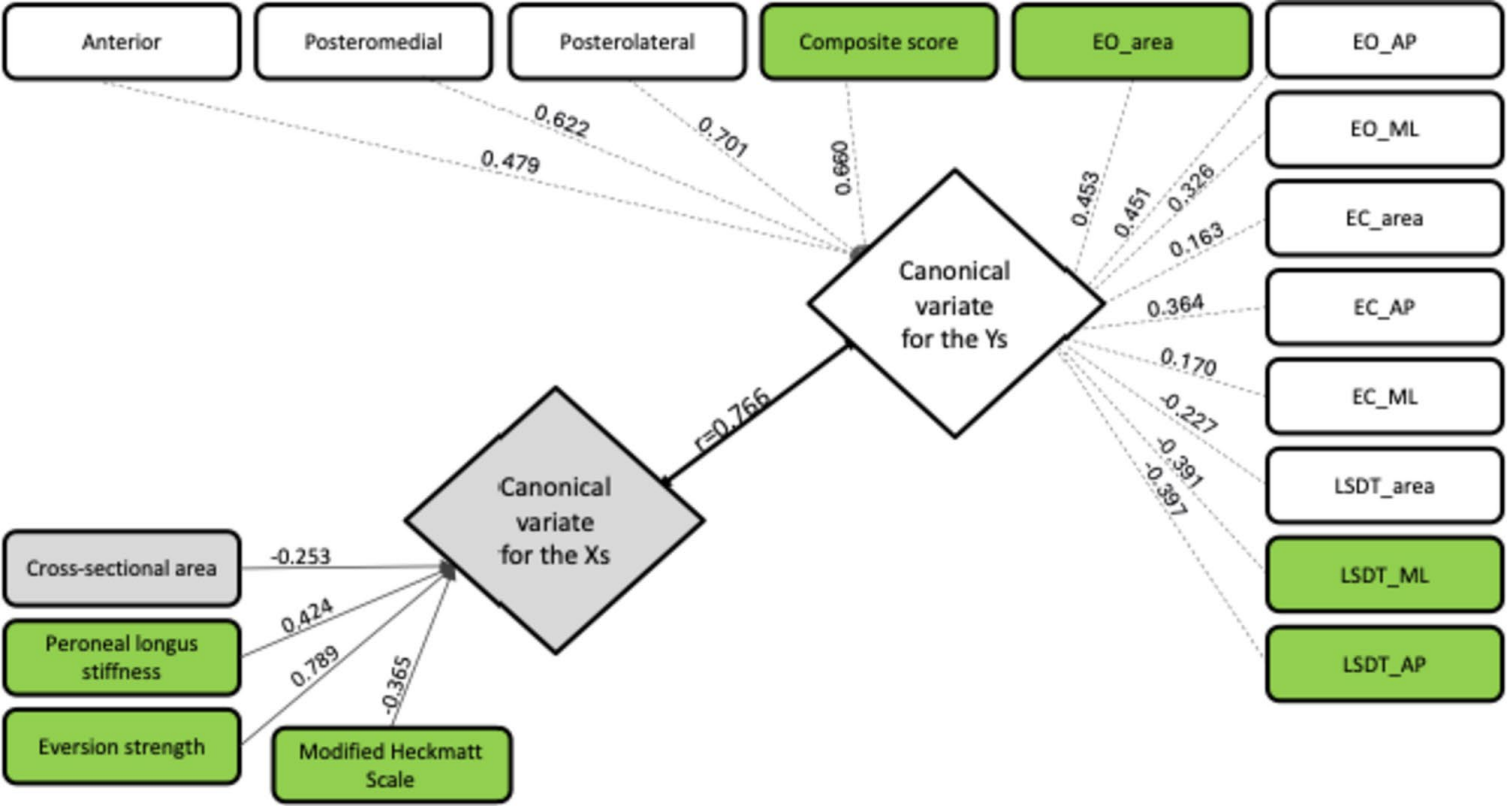
Model depicting the first canonical correlation between peroneal muscle quality and balance functions showing the canonical correlation (r) and the canonical loading for each variable are presented adjacent to their arrow. *Xs* independent variable, *Ys* dependent variable. The significant contributors to the relationship are highlighted in green. *EO* eye open, *EC* eye close, *AP* anterioposterior, *ML* mediolateral, *LSDT* lateral step down test.

The canonical correlation yielded five pairs of canonical variables (Fig. 5). Only the first set had a successful correlation of 0.766 (*p* < 0.001), explaining 25% of the variance of the peroneal muscle quality and 20.1% of the observed static and dynamic balance variances. According to the canonical loading, we have selected eversion strength (rs = 0.789), peroneal longus stiffness (rs = 0.424), and MHS (rs= -0.365) which contributed to *x* (independent factors) in peroneal muscle characteristics. Concurrently, we have selected the total composite score of the YBT (rs = 0.660) to represent the dynamic balance, the area of COP (rs = 0.453) to represent SLST, and anteroposterior excursion sway (rs=-0.397), and mediolateral excursion sway (rs= -0.391) to represent LSDT that have contributed to *y* in balance functions.

According to the stepwise multiple linear regression analysis, the result showed that eversion strength (*p* < 0.001) was associated with the YBT performance regardless of the muscle echogenicity (Table 3). Peroneal longus stiffness (*p* < 0.001) was associated with the YBT performance in the highest grade of muscle echogenicity. Meanwhile, eversion strength was inversely associated with anteroposterior (*p* < 0.001) and mediolateral excursion (*p* = 0.002) sway in moderate echogenicity during the LSDT. Moreover, peroneal longus stiffness was associated with the area of COP during SLST (eyes opened) across different echogenicity severities (*p* < 0.05). Age, gender, and BMI were significantly associated with the different balance functions.

**Table 3:** Stepwise linear regression analysis on the associations between muscle qualities, demographic factors, and balance function.

Variables	Modified Heckmatt cat 2			Modified Heckmatt cat 3			Modified Heckmatt cat 4		
	β	*p*	*R* ^2^	β	*p*	*R* ^2^	β	*p*	*R* ^2^
Y: YBT composite score									
X: BMI	0.603	< 0.001		0.435	< 0.001	0.979	–	–	
X: eversion strength	0.402	< 0.001	0.972	0.346	< 0.001		0.506	< 0.001	
X: age	–	–	–	0.232	0.002		0.167	0.042	0.965
X: peroneal longus stiffness	–	–	–	–	–	–	0.353	< 0.001	
Y: single leg stance test (eyes opened) area of COP									
X: peroneal muscle stiffness	0.721	< 0.001	0.886	0.418	0.008	0.448	0.871	< 0.001	0.749
X: gender	0.308	0.004		0.360	0.021		–	–	–
Y: lateral step down test (anteroposterior sway)									
X: eversion strength	–	–	–	-0.878	< 0.001	0.751	–	–	–
Y: lateral step down test (mediolateral sway)									
X: eversion strength	–	–	–	-0.519	0.002		–	–	–
X: age	–	–	–	-0.382	0.020	0.336	–	–	–
X: BMI	0.425	0.049	0.139	–	–	–	–	–	–

## Discussion

In our study, patients with CAI exhibiting higher muscle echogenicity showed a reduced peroneal muscle size, weaker eversion strength, diminished dynamic stability, and increased postural sway during LSDT compared to those with lower echogenicity. We found that eversion strength significantly predicted YBT performance throughout all echogenicity levels and the sway parameters during the LSDT only in moderate echogenicity. Additionally, higher passive stiffness was positively associated with an area of COP sway in SLST in all grades of echogenicity.

Currently, there are no direct studies on evaluating peroneal muscle echogenicity and balance functions in individuals with CAI. However, Arima et al.^13^ have found an association between an increase in peroneal muscle echogenicity and an increase in sprain frequency in this specific population. According to Mcvey et al.^67^ and Palmieri-Smith et al.^68^, the attributing factors to an increase in adipose tissue within the muscle constitute both reflex inhibition and decreased muscle activity after ankle sprains. Furthermore, the increased muscle adipose tissue could also contribute to poor eversion strength.

Our findings corroborated with a prior study that demonstrated a higher muscle echogenicity and reduced peroneal muscle strength on the affected side among patients with a history of ankle sprains^11^. Apart from diminishing neural activation, the intramuscular adipose tissue or fibrosis could also elicit a systemic effect within the muscles where the decreased inhibitory signal cells from the muscle cells could differentiate the mesenchymal stem cells into fat cells within the muscles^11,69^. The presence of fibro-adipogenic progenitors during muscle injury was suggested as one of the main mechanisms that play a role in the regeneration environment from compensatory to pathologic^70^. The pathologic condition contributes to fatty deposition and fibrotic formation, which affects the muscle quality negatively and physical functions. Furthermore, the increase in intramuscular adipose tissue may affect the electrical signals required for muscle contraction and the decreased ability to fully activate the muscle may impair the patient’s ability to recover from a balance disturbance^22^.

According to Zhu et al.^71^., intramuscular adipose tissue induces various physiological processes within the skeletal muscle.  Intramuscular adipose tissue could affect mitochondrial function, induce muscle inflammation, facilitate fatty infiltration-induced insulin resistance, cause damage to the skeletal muscle fibers and modulate intercellular signalling pathways within the skeletal muscle that regulate myofiber growth and apoptosis^71^. Consequently, this may alter the muscle structure by impairing protein synthesis and decreasing contractile elements in the muscles, resulting in muscle atrophy and decreased neuromuscular activation^71^. In particular, the denervation of motor neurons and muscle fibers^72,73^ coupled with a large reduction in type II muscle fiber, may result in a lower capacity to produce force and impact on neuromuscular activation^22^. Thus, this disruption may lead to a delay in peroneal reaction time, which can influence balance functions and predispose to future ankle sprains. Furthermore, the increase in intramuscular adipose tissue could alter the proprioception through mechanical changes to the muscles and balance functions, such as increasing mechanical impedance and making it harder for the proprioceptors to detect the muscle length and tension^74,75^. Hence, this could impair the sensory feedback pathway and further impair the balance functions^76^.

In addition, our results were also aligned with the findings from other population groups, intramuscular adiposity is associated with accelerated functional impairments^77–80^. For instance, an increase in muscle echogenicity was associated with poor postural control among the elderly, where an accumulation of intramuscular adipose tissue may affect the muscle activation speed and efficiency for maintaining balance^81^. Furthermore, the fat infiltration could trigger muscular inflammation through cell differentiation^82–87^. Thus, this inhibits the ability for muscle repair and regeneration, thereby impairing muscle mechanics and contributing to function deficits.

Our results showed a positive association between eversion strength and the overall YBT performance regardless of echogenicity severity, indicating that a strong eversion strength is required to maintain ankle stability, allowing for more confident and extended reach in all directions of YBT^88^. Our results were aligned with previous studies that have identified the importance of eversion strength lies in its significant effect in stabilising both medial and lateral components of the YBT reach directions^89–91^. These directions may create postural control perturbation. A plausible mechanism could be due to enhanced proprioception required during YBT, where the peroneal muscle received sensory feedback required for increased precision of muscle control around the ankle^9^. Studies comparing the effects of rehabilitation training demonstrated that an increase in eversion strength has better YBT performance, suggesting that changes in muscle echogenicity through training are associated with the change in eversion strength^92–95^. Interestingly, our results did not align with Ko et al.^96^. This could be possibly due to the differences in the characteristics of the patients who require surgical procedures where severe mechanical deficits may have a greater effect on dynamic balance compared to eversion strength^97^. Hence, this may suggest that if prolonged duration of symptoms and muscle disuse were left unattended, this may result in further complications that require non-conservative treatment.

Our findings have demonstrated a negative relationship between eversion strength and anteroposterior and mediolateral sway demonstrated during LSDT, especially among CAI patients who have moderate echogenicity in peroneal muscles. It was suggested that poor eversion strength may impede proper foot positioning during lateral movement and the weight-bearing phase of this test^98^. Hence, this results in postural perturbance and potentially causes an increased medial knee displacement or loss of balance^99^. A plausible suggestion that the association was observed only in the moderate echogenicity of the peroneal muscle denotes a critical threshold where eversion strength may be compromised and impaired postural control is observed. Conversely, the absence of associations in other echogenicity levels indicates a complex interplay of factors influencing eversion strength and postural control, potentially involving proprioceptive and neuromuscular adaptations. For instance, patients with heightened echo intensity may experience severe muscle inhibition and joint instability, which result in adapting to a compensatory mechanism, leading to a compensatory mechanism that may overshadow the role of eversion strength^100^. In contrast, the lack of association between eversion strength and postural sway during LSDT among patients with mild muscle echogenicity suggests that muscle composition is not severely altered and neuromuscular functions are well-preserved^65^. Currently, our study is the first that has evaluated the relationship between peroneal muscle quality and postural sway displacement during LSDT. Hence, we were not able to compare our findings with other previous works. Previous studies mainly evaluated the quality evaluation of the LSDT in both healthy subjects and CAI individuals^101–104^. Only one study has investigated the postural control parameters during LSDT. However, the relationship between peroneal muscle quality and postural control parameters during LSDT was not evaluated^54^. Furthermore, the authors did not identify any significant results on the displacement of postural sway parameters between CAI and the healthy control group^54^. Therefore, future studies should explore the COP displacement related to movement quality and clinical outcomes in the context of LSDT. This approach may elucidate the underlying mechanisms for poor postural control during unilateral weight-bearing activities.

In addition, our findings demonstrated that an increase in peroneal muscle stiffness is associated with (1) a longer reach distance in the overall total composite score of the YBT in the highest grade of echogenicity, (2) an increased area of COP excursion sway during SLST irrespective of muscle echogenicity among CAI patients. Our findings are consistent with previous studies that assessed passive stiffness in individuals with CAI and ankle sprains using myotonometry^27,105^.The observed increase in passive stiffness of the peroneal muscles is a compensatory mechanism for the reduced stiffness of the ankle joint following lateral ankle sprains (LAS)^106–108^. This increased stiffness is primarily attributed to muscle dysfunction resulting from elevated fat infiltration or fibrosis, which replaces contractile tissue and disrupts fiber alignment^109^.

The force contribution of the peroneal muscles may reach a saturation point at low to moderate levels of motor unit recruitment, potentially hindered by fat infiltration^27,110–114^. Therefore, enhanced passive stiffness has been identified as an effective medium for transmitting movement-generated mechanical information and facilitating tension transfer among interconnected tissues^115^. However, individuals with heightened muscle stiffness may need to compensate through muscle overactivation to maintain stability in a unipodal stance, which can impair coordinated motor actions and increase the risk of coordination deficits and injury^116^.

According to Theret et al.^117^, muscle fibrosis resulting from sprains may hinder muscle regeneration by promoting the differentiation of fibro-adipogenic progenitors into fibroblasts and adipocytes instead of myogenic cells. This process can lead to increased muscle stiffness and reduced flexibility. Elevated stiffness may also impair neuromuscular control by restricting the range of motion and the dynamics of muscle contractions^117^. Furthermore, evidence from healthy populations suggests that increased passive stiffness may result in fascial rigidity, which can hyperstimulate muscle spindles and decrease muscle compliance, thus impeding range of motion and overall mobility^118^ This limits the body’s ability to adapt to sudden changes and perturbations, and the inability to balance during dynamic activities. Furthermore, increasing peroneal muscle stiffness may reduce the body’s ability to absorb and dissipate energy during sudden movement, resulting in a less adaptative postural control strategy^119^. Therefore, our results may shed some light on how intramuscular adipose tissue is associated with the changes in the mechanics of the skeletal muscle and impacts the balance functions. However, given that the stiffness measurements are restricted to the probe region and stiffness is not uniform across the entire muscle, the implication may be limited^120^. Future studies should investigate various clinical methods to evaluate passive stiffness.

Our study had several limitations. Firstly, due to the cross-sectional design, the sequence changes for the balance function deficit or the presence of poor peroneal muscle quality remain unclear, without being able to establish causality. Longitudinal studies should be conducted to establish causality. Secondly, the convenient sampling method from the hospital could lack the representativeness of the population and may not be generalised to other population groups such as among the elderly and athletes, especially when both population groups have a higher exposure to repeated ankle sprains. As such, studies should be stratified in future recruitment to reduce sampling bias. Thirdly, the study also recruited a greater proportion of female participants compared to males. However, since CAI was suggested to be significantly higher in females than males^121^, our results were likely to represent the general population and provide a better understanding. Future studies should investigate the potential moderating effect of gender. Lastly, the modified Heckmatt scale to evaluate muscle echogenicity is a qualitative assessment. In our study, the usage of the modified Heckmatt scale was conducted independently by a registered physiotherapist and the researcher to reduce subjectivity. Future studies should complement an objective measurement such as magnetic resonance imaging or muscle biopsy which is more invasive to measure muscle echogenicity.

## Conclusion

In conclusion, participants with increased peroneal muscle echogenicity exhibited poorer outcomes, including peroneal muscle atrophy, diminished eversion strength, and compromised balance functions in patients with CAI. Future rehabilitation programmes should concentrate on enhancing peroneal muscle architecture as a potential therapeutic strategy. In particular, biophysical interventions such as neuromuscular electrical stimulation, pulse-electromagnetic field therapy, or whole-body vibration therapy etc. aimed at improving peroneal muscle activity to restore the architecture and quality of the peroneal muscle and may potentially aid in the enhancement of balance functions.

## Electronic supplementary material

Below is the link to the electronic supplementary material.

Supplementary Material 1

Supplementary Material 2

## Data Availability

The datasets used and/or analysed during the current study are available from the corresponding author on reasonable request.

## Abbreviations

BMI: Body mass index
CAI: Chronic ankle instability
CAIT: Cumberland ankle instability tool
COP: Centre of pressure
CSA: Cross-sectional area
SLST: Single leg stance test
LAS: Lateral ankle sprain
LSDT: Lateral step down test
YBT: Y balance test
