# Supplementary material for: A cross-sectional study on peroneal muscle echogenicity changes and their effects on balance functions in individuals with chronic ankle instability
Source: Sci Rep. 2025 Apr 29;15:15090. doi: 10.1038/s41598-025-00175-3 (PMC12041604; doi:10.1038/s41598-025-00175-3)
Supplement: Supplementary file 1 — Supplementary Material 1 [file 41598_2025_175_MOESM1_ESM.pdf]

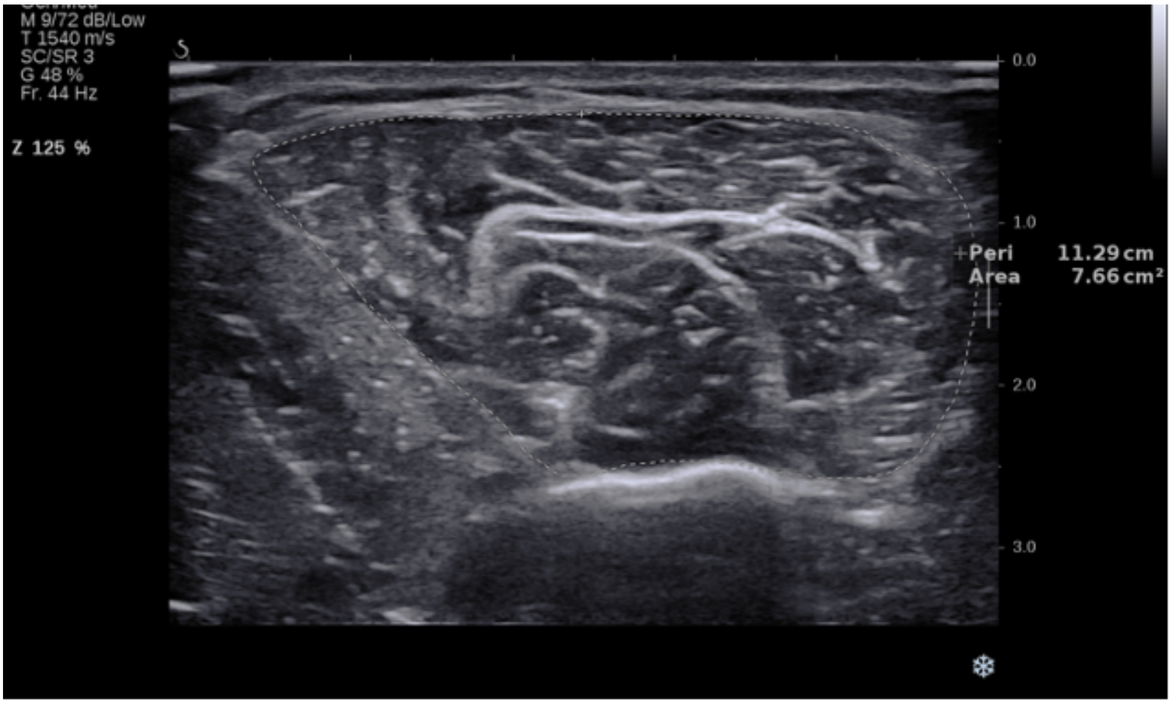

APPENDIX 1 cross-sectional area of the peroneal muscle

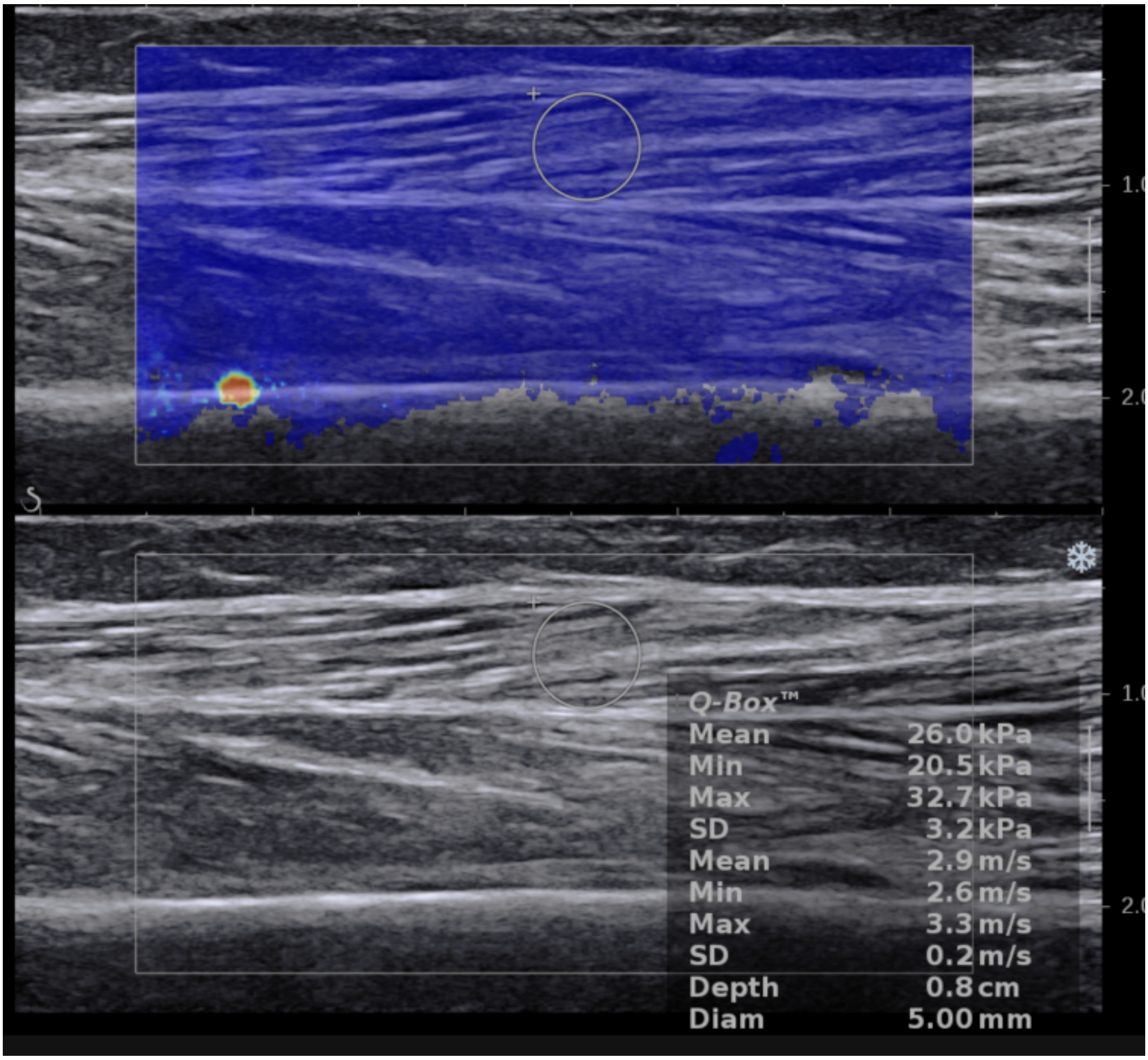

APPENDIX 2 Example of a shearwave elastography of the peroneal longus
