# Supplementary material for: A cross-sectional study on peroneal muscle echogenicity changes and their effects on balance functions in individuals with chronic ankle instability
Source: Sci Rep. 2025 Apr 29;15:15090. doi: 10.1038/s41598-025-00175-3 (PMC12041604; doi:10.1038/s41598-025-00175-3)
Supplement: Supplementary file 2 — Supplementary Material 2 [file 41598_2025_175_MOESM2_ESM.docx]

**Appendix**

Table 2 VIF factors for dependent variables

|  |  | **VIF** | | |
| --- | --- | --- | --- | --- |
| **Dependent variable** | **Independent variables** | **MHS grade 2** | **MHS grade 3** | **MHS grade 4** |
| YBT composite score |  |  |  |  |
|  | Eversion strength | 1.655 | 1.079 | 1.181 |
|  | Passive Stiffness | 1.135 | 1.088 | 1.191 |
|  | Age | 1.157 | 1.043 | 1.419 |
|  | Gender | 1.117 | 1.276 | 1.207 |
|  | BMI | 1.759 | 1.294 | 1.374 |
| SLST_area of COP |  |  |  |  |
|  | Eversion strength | 1.655 | 1.079 | 1.056 |
|  | Passive Stiffness | 1.135 | 1.088 | 1.281 |
|  | Age | 1.157 | 1.043 | 1.389 |
|  | Gender | 1.117 | 1.276 | 1.094 |
|  | BMI | 1.759 | 1.294 | 1.351 |
| LSDT_mediolateral and anteriorposterior excursion sway |  |  |  |  |
|  | Eversion strength | 1.655 | 1.040 | 2.570 |
|  | Passive Stiffness | 1.135 | 1.098 | 3.376 |
|  | Age | 1.157 | 1.101 | 1.271 |
|  | Gender | 1.117 | 1.253 | 2.333 |
|  | BMI | 1.759 | 1.196 | 2.384 |
